# Microbial and Chemical Profiles of Commercial Kombucha Products

**DOI:** 10.3390/nu14030670

**Published:** 2022-02-05

**Authors:** Jieping Yang, Venu Lagishetty, Patrick Kurnia, Susanne M. Henning, Aaron I. Ahdoot, Jonathan P. Jacobs

**Affiliations:** 1 Department of Medicine, David Geffen School of Medicine at UCLA, Los Angeles, CA 90095, USA; jiepingyang@mednet.ucla.edu (J.Y.); vlagishetty@gmail.com (V.L.); patrickt75@g.ucla.edu (P.K.); shenning@mednet.ucla.edu (S.M.H.); aaronahdoot@ucla.edu (A.I.A.); 2 The Vatche and Tamar Manoukian Division of Digestive Diseases, David Geffen School of Medicine at UCLA, Los Angeles, CA 90095, USA; 3 Division of Gastroenterology, Hepatology and Parenteral Nutrition, Veterans Affairs Greater Los Angeles Healthcare System, Los Angeles, CA 90073, USA

**Keywords:** kombucha, bacteria, yeast, metagenome, metabolome, tea polyphenols, antioxidants

## Abstract

Kombucha is an increasingly popular functional beverage that has gained attention for its unique combination of phytochemicals, metabolites, and microbes. Previous chemical and microbial composition analyses of kombucha have mainly focused on understanding their changes during fermentation. Very limited information is available regarding nutrient profiles of final kombucha products in the market. In this study, we compared the major chemicals (tea polyphenols, caffeine), antioxidant properties, microbial and metabolomic profiles of nine commercial kombucha products using shotgun metagenomics, internal transcribed spacer sequencing, untargeted metabolomics, and targeted chemical assays. All of the nine kombucha products showed similar acidity but great differences in chemicals, metabolites, microbes, and antioxidant activities. Most kombucha products are dominated by the probiotic *Bacillus coagulans* or bacteria capable of fermentation including *Lactobacillus nagelii*, *Gluconacetobacter*, *Gluconobacter*, and *Komagataeibacter* species. We found that all nine kombuchas also contained varying levels of enteric bacteria including *Bacteroides thetaiotamicron*, *Escherischia coli*, *Enterococcus faecalis*, *Bacteroides fragilis*, *Enterobacter cloacae complex*, and *Akkermansia muciniphila*. The fungal composition of kombucha products was characterized by predominance of fermenting yeast including *Brettanomyces* species and *Cyberlindnera jadinii*. Kombucha varied widely in chemical content assessed by global untargeted metabolomics, with metabolomic variation being significantly associated with metagenomic profiles. Variation in tea bases, bacteria/yeast starter cultures, and duration of fermentation may all contribute to the observed large differences in the microbial and chemical profiles of final kombucha products.

## 1. Introduction

Kombucha is a fermented tea drink commonly consumed for its potential health benefits [1]. The fermentation occurs by providing Symbiotic Culture of Bacteria and Yeasts (SCOBY), a biofilm of cellulose containing the bacteria and yeasts, as a starter to sugary tea [2,3]. Kombucha yeast have been reported to include members of the *Zygosaccharomyces* and *Brettanomyces* genera [4]. Bacteria reported in kombucha cultures include acetic acid bacteria (*Gluconacetobacter* and *Acetobacter*) and lactic acid bacteria (*Lactobacillus*) [3,5]. The extensive interactions between bacteria and yeasts feeding on sugar, tea base as well as other added substrates lead to the production of a wide range of bioactive metabolites including vitamins, amino acids, and ethanol [5]. 

The most commonly used teas for fermentation are black tea, white tea and green tea [6]. The chemical composition of tea has been well documented and a variety of factors, such as storage and manufacturing process, determine the tea chemical composition [7]. Tea polyphenols constitute up to 30% of the green tea dry weight and are known for their health benefits [8,9,10]. Catechins are the characteristic components of tea polyphenols, including catechin (C), epicatechin (EC), epigallocatechin (EGC), and epigallocatechin gallate (EGCG) [11]. During the production of black tea, about 75% of catechins undergo enzymatic transformation consisting of oxidation and partial polymerization into thearubigins [9,12]. The unique chemical profiles of each tea base are likely to be reflected in the final chemical composition of kombucha and may lead to different biological activities [13,14,15].

There has been great interest in fermented food products, including kombucha, as a source of probiotics (i.e., live microbes with beneficial effects on health). Potential beneficial properties of probiotics including inhibition of pathogenic bacteria, promotion of health-associated gut microbial communities, interaction with the intestinal epithelium, metabolism of certain nutrients, and modulation of signaling within the immune system [16]. Many existing purported probiotics belong to the *Lactobacillus* genus, which are often present in kombucha [17]. To satisfy the claim of probiotics, several probiotics are often added to commercial kombucha products. *Bacillus coagulans*, a lactic acid-producing bacteria resistant to high temperature, has been one of the most common [18]. Other probiotics that are added to commercial kombucha products include *Bacillus subtilis*, *Saccharomyces boulardii*, and *Lactobacillus rhamnosus* [1].

The complex interactions among the different combination of yeast/bacteria, tea base, manufacturing process, and probiotic addition create the potential for a wide diversity of bacterial and fungal compositions, tea polyphenol levels, and metabolite contents of kombucha products [3,19,20]. Such variation could greatly impact the overall health benefits of kombucha. In this study, we investigated heterogeneity among a sample of commercial kombucha products from a multi-omics perspective. For this purpose, we employed chemical, metabolomics and metagenomics analyses to understand the diversity of kombucha by profiling nine commercial kombucha products.

## 2. Materials and Methods

### 2.1. Kombucha Samples

Nine different commercial kombucha products (*n* = 3 samples per product), either in original or ginger flavor to minimize differences in added ingredients, were purchased from three supermarkets in the Los Angeles metropolitan area on 2/2/21: GT’s Synergy Gingerade (A1), GT’s Classic Original (A2), Better Booch Ginger Boost (B), Bottled Brew Dr. Ginger Lemon (C1), Canned Brew Dr. Ginger Lemon (C2), Health Ade Ginger Lemon (D), Humm Kombucha Ginger (E1), Humm Zero Kombucha Ginger Lemonade (E2), and Kevita Master Brew Ginger Kombucha (F). These kombucha products were selected based upon the high market share of these six manufacturers, which together represent an estimated 86% of commercial kombucha sales in the United States in 2021 [21]. As samples for each kombucha product came from the same batch, this analysis could not assess for differences across batches of the same kombucha product. Ingredient labels and nutritional information provided by each manufacturer are shown in Table 1. The kombucha product labels indicated a range of energy values from 3 to 17 kcal/100 g; use of black tea, green tee, or both; additional probiotic cultures in some products; and various flavoring ingredients. Within three days of purchase, the contents of each bottle or can were carefully mixed so that no visible sediment remained. The mixed kombucha products were then aliquoted into 50 mL conical tubes and centrifuged at 1300× *g* for 10 min. The supernatant was separated from the pellets and both were kept frozen at −80 °C until assays were performed.

### 2.2. Levels of Tea Catechins and Caffeine Analyzed by High Performance Liquid Chromatography (HPLC)

The kombucha supernatant samples were centrifuged at 14,000 rpm for 10 min and the supernatants were used for the analysis. Analysis was performed on a Waters 2690 HPLC equipped with photodiode array detector. Chromatographic separation was achieved on a Zorbax SB-C18 (4.6 × 250 mm 5 um Agilent, Santa Clara, CA, USA) column and the column temperature was held at 35 C. Eluent A consisted of 0.1% phosphoric acid in water and eluent B consisted of 0.1% phosphoric acid in acetonitrile. Gradient elution was performed with the 95% A to 70% A in 40 min with flow rate of 0.75 mL/min. Catechins and caffeine concentration were determined at 280 nm.

### 2.3. Gallic Acid Equivalent (GAE) Assay

GAE assay was performed as previously described using the Folin–Ciocalteau reagent [22]. The reading was performed at 755 nm in a ThermoMax microplate reader (Molecular Devices, Sunnyvale, CA, USA) at room temperature. Standard curves were used to convert the average absorbance of each sample into mg/g GAE.

### 2.4. Trolox Equivalent Antioxidant Capacity (TEAC) Assay

TEAC was performed as previously described [23]. Kombucha supernatant samples were centrifuged at 4000 rpm for 15 min at 4 °C and the supernatants were transferred into a clean tube and diluted in Na/K buffer (pH 7.0). The Kombucha sample absorbance readings (750 nm) were in the linear range of Trolox standard curve. Total antioxidant activity was calculated using a Trolox (Sigma-Aldrich, St. Louis, MO, USA) standard curve with a concentration range of 0 to 300 µM and was expressed in Trolox equivalents. All samples were performed in triplicate and final TEAC result was calculated as Trolox equivalents (mg) per L.

### 2.5. Ethanol Content ANALYZED by Gas Chromatography (GC)

The kombucha supernatant samples were centrifuged at 14,000 rpm for 10 min and the supernatants were diluted with Milipore water (1:1). The ethanol content in diluted kombucha supernatants was quantified by GC FID (Agilent 7890A) and RTX-Stabilwax capillary column (Restek, Bellefonte, PA, USA). Ethanol concentration was determined by comparing the peak area and retention with the ethanol standard peak area and retention time.

### 2.6. pH Measurements

The pH of each product was measured using a Cole-Parmer P200 pH meter. Each measurement was replicated three times.

### 2.7. Shotgun Metagenomics Analysis

Microbial DNA was extracted from frozen pellets using the Qiagen Powersoil Pro kit with bead beating according to the manufacturer’s protocol. DNA libraries were then prepared using the Celero PCR Workflow with Enzymatic Fragmentation DNA-Seq library preparation kit (Cat # 9363; NuGEN Technologies, Inc., San Carlos, CA, USA) according to the manufacturer’s protocol. Briefly, 200 ng of extracted DNA was enzymatically fragmented followed by adapter ligation, amplification and purification. Library concentrations were measured by Qubit and size distribution was assessed by Bioanalyzer. Purified libraries were diluted to 10 nM and pooled for 2 × 150 paired-end sequencing using the Illumina Novaseq 6000 sequencing system (S4 flowcell). Mean sequence depth for the kombucha samples was 24.8 million paired-end reads per sample (range 9.7–68.1 million). Read-level quality control was performed using KneadData with default settings [24]. Taxonomic annotations were made using MetaPhlAn 3.0, which uses clade-specific markers to identify microbial species present in a sample and their relative abundances. Alpha diversity was assessed using the Shannon index implemented in the R phyloseq package [25]. Beta diversity was calculated using Bray–Curtis dissimilarity and visualized by principal coordinates analysis using the vegan package in R. Functional annotation was performed using HUMAnN 3.0 with the UniRef90 database. Microbial gene abundances were collapsed into functional pathways defined by MetaCyc annotations.

### 2.8. ITS Sequencing

The fungal internal transcribed spacer (ITS) region was amplified from extracted DNA samples using the ITS1f and ITS2 primers according to a published protocol [26]. Amplified product was then pooled and sequenced using an Illumina MiSeq (250 × 2 bp sequencing, v2 kit). DADA2 was used to perform primer trimming, quality-filtering, inference of amplicon sequence variants (ASV), chimera removal, and taxonomy assignment based on the UNITE database [27]. Mean sequence depth after processing was 312,677 paired-end sequences per sample (range 23,664–472,429). Alpha and beta diversity were assessed as was described for shotgun metagenomics data.

### 2.9. Metabolomics

Metabolomics analysis of kombucha supernatant samples was performed by The Metabolomics Innovation Center (Edmonton, Canada). The workflow included sample pre-treatment and normalization, chemical isotope labeling (CIL), LC-MS analysis, data processing and metabolite identification. In the CIL LC-MS metabolome analysis, the whole metabolome was analyzed by targeting four submetabolomes or channels: amine/phenol-, carboxyl-, hydroxyl- and carbonyl-submetabolome.^1^ The combined results from four channels could provide comprehensive profile of the entire metabolome, e.g., about 86% to 96% of the chemical space in various metabolome databases.^1^ In each channel, after sample pre-treatment step (e.g., protein precipitation, metabolite extraction, etc.), all samples were first derivatized with a pair of isotopic labeling reagents (i.e., ^12^C-/^13^C-reagents) prior to LC-MS analysis. Individual samples were labeled with ^12^C-reagents and a pooled sample, which was generated by mixing an aliquot from each individual sample, was labeled with ^13^C-reagents. After labeling, the ^12^C-labeled individual sample was mixed with the same amount of the ^13^C-labeled pool, followed by LC-MS analysis. In the mass spectra, each metabolite was detected as a peak pair, i.e., the light peak from the ^12^C-labeled individual sample and the heavy peak from the ^13^C-labeled pool. The peak intensity ratio between ^12^C-peak and ^13^C-peak represents the relative quantification result for a specific metabolite in an individual sample. Since the same ^13^C-labeled pooled sample was spiked into all the ^12^C-labeled individual samples, the ^12^C-/^13^C-peak ratio values of a specific metabolite in all the individual samples reflected the concentration differences of the metabolite in these samples. The ^13^C-labeled pooled sample served as an internal reference for analyzing all individual samples to correct for matrix and ion suppression effects as well as any instrument sensitivity drifts for accurate and precise quantification. All LC-MS analysis were carried out on using Agilent 1290 LC linked to Bruker Impact II QTOF Mass Spectrometer. The column used was Agilent eclipse plus reversed-phase C18 column (150 × 2.1 mm, 1.8 µm particle size) and the column oven temperature was 40 °C. Mobile phase A was 0.1% (*v*/*v*) formic acid in water and mobile phase B was 0.1% (*v*/*v*) formic acid in acetonitrile. The gradient setting was: t = 0 min, 25% B; t = 10 min, 99% B; t = 15 min, 99% B; t = 15.1 min, 25% B; t = 18 min, 25% B. The flow rate was 400 µL/min. Mass spectral acquisition rate was 1 Hz, with an m/z range from 220 to 1000.

Peaks were annotated using a three-tier approach. In tier 1, peak pairs were searched against a labeled metabolite library (CIL Library, contains more than 1500 entries) based on accurate mass and retention time. 251 peak pairs were positively identified in this manner. In tier 2, linked identity library (LI Library) was used for putative identification of the remaining peak pairs. The LI Library includes over 9000 pathway-related metabolites, providing high-confidence putative identification results based on accurate mass and predicted retention time matches. 1090 peak pairs were putatively identified by this approach. In tier 3, the remaining peak pairs were searched, based on accurate mass match, against the MyCompoundID (MCID) library composed of 8021 known human endogenous metabolites (zero-reaction library), their predicted metabolic products from one metabolic reaction (375,809 compounds) (one-reaction library) and two metabolic reactions (10,583,901 compounds) (two-reaction library). 1197, 2976 and 748 peak pairs were matched in the zero-, one- and two-reaction libraries, respectively. In total, 7459 unique peak pairs were detected, of which 1341 were identified with high-confidence (tiers 1 and 2) and the majority of the remaining peaks had putative annotations. 

Global differences in metabolomics profiles were visualized by performing principal coordinates analysis of Euclidean distances. Sparse partial least-squares discriminant analysis (sPLS-DA) implemented in the mixOmics package in R was used to select a limited number of annotated metabolites that differentiate kombucha samples [28]. A tuning process was performed to select the optimal number of metabolites for each of two components (X-variates) to minimize the balanced error rate in 5-fold validation.

### 2.10. Statistical Analysis

Data distributions were summarized by both mean and SD as well as by median, minimum, and maximum (Supplementary Table S1). Normality of distribution was assessed by the Shapiro–Wilk test. Significance of differences across samples in measured chemicals and alpha diversity was assessed by ANOVA in R using the built-in aov function. Upon finding significant differences across all products, we used Tukey’s Post-hoc Test to perform pairwise comparisons between each kombucha product. For measures in which one or two out of the nine groups showed a significant p-value by the Shapiro–Wilk test, we generated additional figures showing distributions as boxplots with significance determined by Kruskal–Wallis with post-hoc Dunn’s test (Supplementary Figure S1). 

Significance of global differences across kombucha products (beta diversity) in bacterial composition, fungal composition, microbial gene abundances, and metabolomics profiles was determined using multivariate Adonis in the R package vegan. Metagenomics data (UniRef genes) and metabolomics data were superimposed using Procrustes and significance was assessed using the Mantel test implemented in the vegan package in R. MetaCyc pathways and metabolites that were differentially abundant across kombucha products were identified using Kruskal–Wallis. *p*-values for differential abundance were adjusted using the Benjamini–Hochberg method to correct for multiple hypothesis testing (*p* < 0.05 for significance). Partial Spearman correlations adjusting for kombucha product were calculated for all pairwise combinations of differentially abundant pathways and metabolites using the PResiduals package in R. Correlations were adjusted by the Benjamini–Hochberg method to control false discovery rate (FDR) at <0.1. 

## 3. Results

### 3.1. Microbial Composition

**Figure 1:**
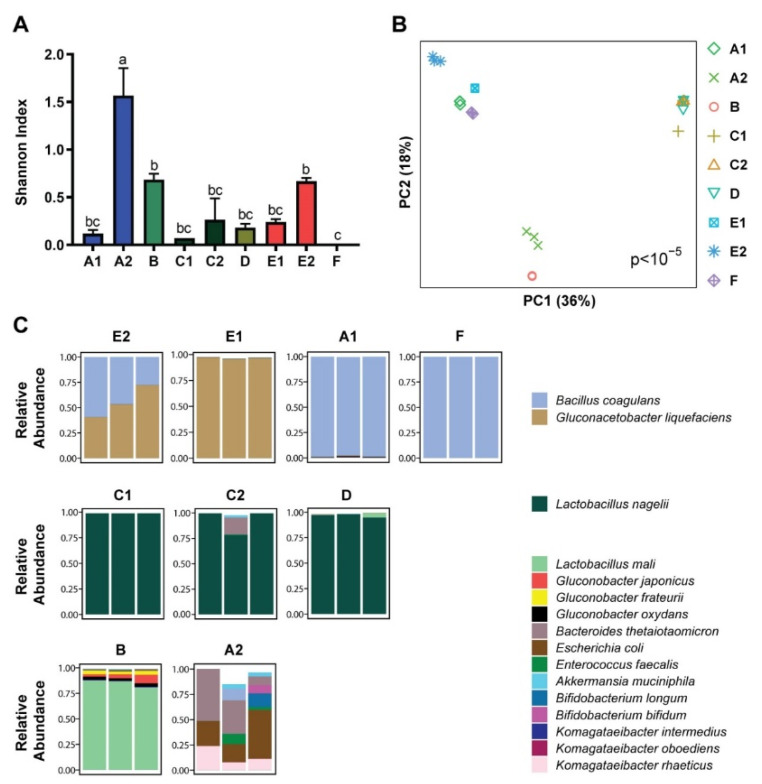
Kombucha products vary greatly in microbial composition and diversity. (**A**) Microbial alpha diversity as measured by the Shannon index of richness and evenness is shown for each sample, grouped by kombucha product. Means in a column without a common letter differ; *p* < 0.05. Product C2 had *p* < 0.05 by the Shapiro–Wilk test of normality. (**B**) Principal coordinates analysis plot visualizing microbial beta diversity by Bray–Curtis dissimilarity. Each symbol represents a sample; kombucha product is indicated by symbol color and shape. Significance of differences across products was assessed by Adonis. (**C**) Taxa summary plots showing the relative abundances of the 16 most abundant bacterial species across the samples. Each bar represents one sample; samples are grouped by kombucha product. Color indicates species. The bars do not necessarily add to 1 as lower abundance microbes are not shown.

**Figure 2:**
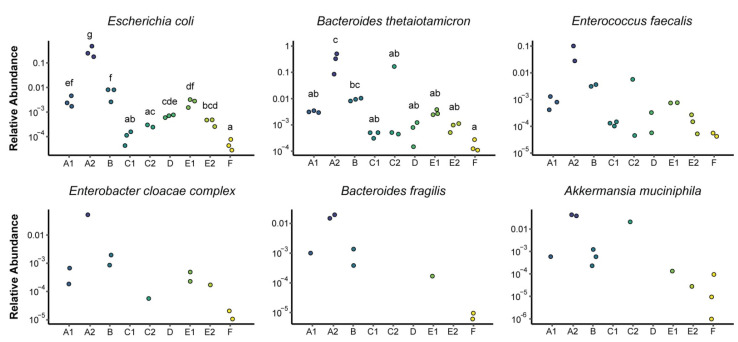
Relative abundances of enteric bacteria detected in kombucha. Each dot represents one sample with detectable levels of the indicated enteric bacteria. Significance of differences in two widely detected enteric bacteria, *E. coli* and *B. thetaiotamicron*, was determined by ANOVA with post-hoc Tukey. Means in a column without a common letter differ; *p* < 0.05.

Shotgun metagenomics sequencing was performed of three samples each from nine commercial kombucha products shown in Table 1. The initial analysis focused on bacterial composition at the species level based on annotation of shotgun reads against a reference database. Most kombucha products were found to have low bacterial diversity due to dominance by a single bacterium, among which A2 showed significantly higher diversity compared to all other products and F showed significantly lower diversity (Figure 1A). Bacterial composition was strongly associated with kombucha product (*p* < 10^−5^). In all cases, the three samples from each product tightly clustered with one another (Figure 1B). The nine kombucha products formed three groups according to their dominant bacteria (Figure 1B,C). The first group (E1, E2, A1, F) was characterized by predominance of *Bacillus coagulans* and/or *Gluconacetobacter liquefaciens*. The second group (C1, C2, D) was characterized by predominance of *Lactobacillus nagelii*. One of the remaining kombucha products (B) was characterized by high abundance of *Lactobacillus mali* and presence of *Gluconobacter* species while the other (A2) included both fermenting bacteria in the *Komagataeibacter* genus (primarily *K. rhaeticus*) as well as large populations of gut-derived bacteria. *E. coli* and *Bacteroides thetaiotamicron* together represented the majority of bacteria detected in all three samples of A2. Given the high abundances of enteric bacteria found in this kombucha product, we assessed whether these bacteria could also be detected in the other eight kombucha products. All were found to contain enteric bacteria, especially *Bacteroides thetaiotamicron*, *E. coli*, and *Enterococcus faecalis*, as well as less commonly other species including *Enterobacter cloacae complex*, *Bacteroides fragilis* and *Akkermansia muciniphila* (Figure 2). A2 had significantly higher levels of *E. coli* and *B. thetaiotamicron* than the other products while F had significantly lower levels of these microbes.

**Figure 3:**
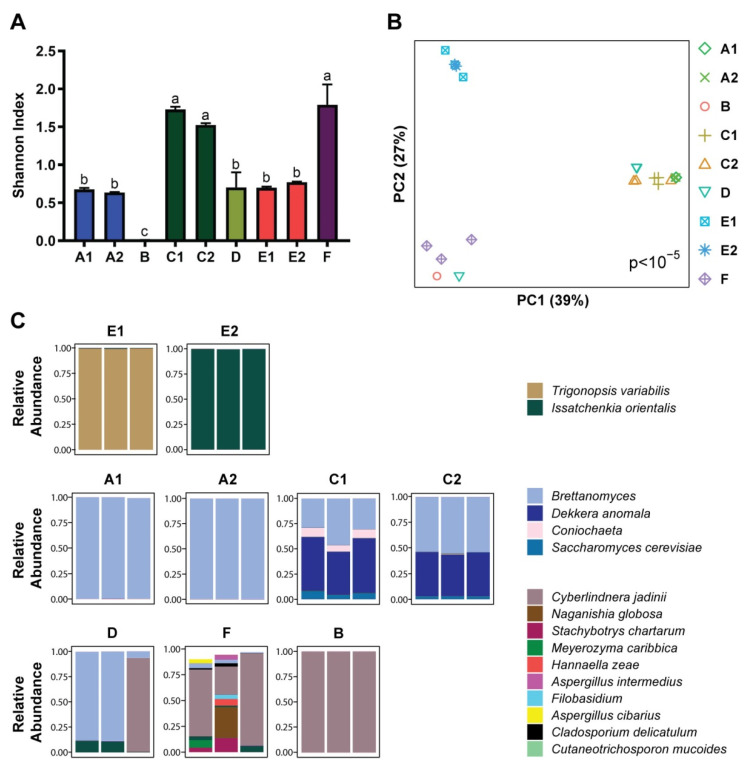
Fungal diversity and composition vary across kombucha products. (**A**) Fungal alpha diversity as measured by the Shannon index of richness and evenness is shown for each sample, grouped by kombucha product. Means in a column without a common letter differ; *p* < 0.05. Product D had *p* < 0.05 by the Shapiro–Wilk test of normality. (**B**) Principal coordinates analysis plot visualizing fungal beta diversity by Bray–Curtis dissimilarity. Each symbol represents a sample; kombucha product is indicated by symbol color and shape. Significance of differences across products was assessed by Adonis. (**C**) Taxa summary plots showing the relative abundances of the 16 most abundant fungal species across the samples. Each bar represents one sample; samples are grouped by kombucha product. Color indicates species. In some cases, the species was uncharacterized, in which case only the genus name is shown. The bars do not necessarily add to 1 as lower abundance fungi are not shown.

The reference database used to annotate shotgun metagenomics sequence data contained over 500 fungal genomes, but of these, only *Saccharomyces cerevisiae* was detected in the kombucha samples. Given the paucity of detected kombucha yeast genomes, we assessed the fungal composition of kombucha by ITS sequencing. Kombucha products varied in fungal diversity with a pattern that differed from that of bacterial diversity (Figure 3A). For instance, the kombucha product with lowest bacterial diversity (F) had the highest fungal diversity. Fungal composition was strongly associated with kombucha product (*p* < 10^−5^) (Figure 3B). The nine kombucha products had similar yeast composition across the three samples of each product with the exception of a single product (D). Most kombucha products showed predominance of one or two yeast species and grouped together based on manufacturer and the dominant yeast (Figure 3B,C). The largest group (A1, A2, C1, C2, and two samples of D) showed predominance of an uncharacterized species of *Brettanomyces* (a yeast genus associated with wine fermentation) and a related yeast, *Dekkera anomala* (a member of the *Brettanomyces* genus). A second group (B, F, and one sample of D) was characterized by predominance of *Cyberlindnera jadinii*. The remaining two kombucha products (E1, E2) grouped together and were characterized by predominance of *Trigonopsis variabilis* or *Issatchenkia orientalis*.

### 3.2. Metabolomics and Metagenomics Profiles

**Figure 4:**
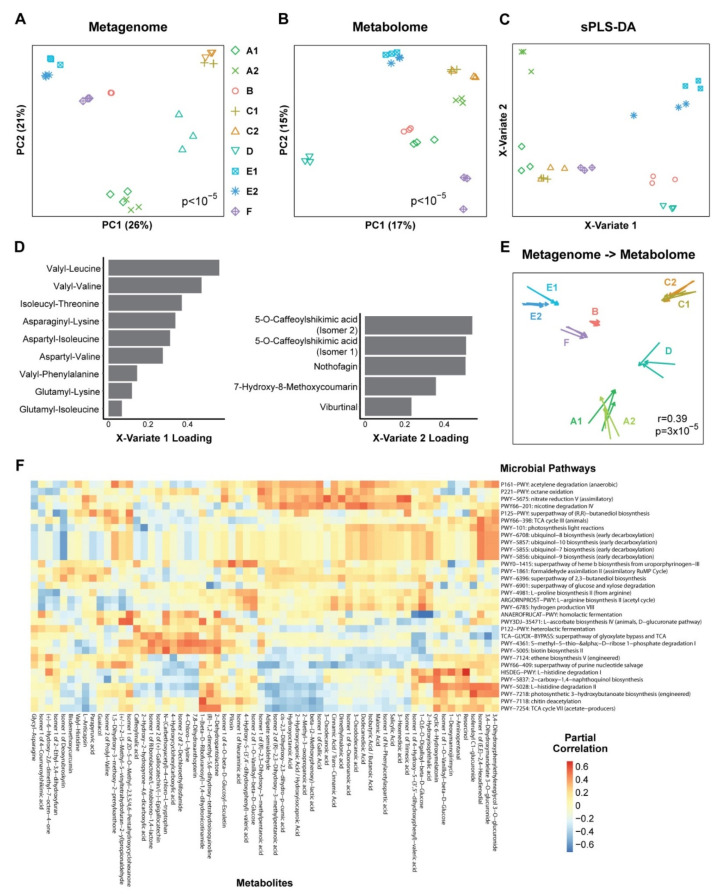
Differences in metabolites and microbial gene content across kombucha products. (**A**,**B**) Principal coordinates analysis plots representing (**A**) microbial gene content (“metagenome”) and (**B**) metabolomics profiles across the kombucha samples, with symbol/color indicating kombucha product. Significance of differences across products was assessed by Adonis. (**C**) Sparse partial least squares discriminant analysis (sPLS-DA) was used to visualize kombucha products in a supervised manner based upon two derived axes (X-variates 1 and 2) containing 9 and 5 metabolites, respectively. (**D**) Loadings of the metabolites contributing to X-variates 1 and 2 derived from sPLS-DA. (**E**) Procrustes analysis superimposing microbial gene abundances and metabolomics profiles. Arrows in the Procrustes plot point from the gene content data (“Metagenome”) to the metabolomics data (“Metabolome”). Significance of correlations between the two data sets was determined by the Mantel test with 100,000 permutations. (**F**) Heat map depicting partial correlations between MetaCyc pathways and metabolites that were differentially abundant across kombucha products. Correlations were adjusted for kombucha product and are only shown for pathways and metabolites that had at least one partial correlation with FDR <0.1.

We then used the shotgun metagenomics data to assess the functional potential of kombucha microbes based on UniRef90 annotation. Gene content was strongly associated with kombucha product (*p* < 10^−5^), with the three samples of each product clustering together (Figure 4A). When summarized at the pathway level, 62 MetaCyc pathways were found to be differentially abundant across kombucha products. Global untargeted metabolomics was then performed to compare chemical composition of the nine kombucha products. Metabolite profiles were strongly associated with kombucha product (*p* < 10^−5^) and differential abundance testing demonstrated 1257 positively or putatively identified metabolites that significantly differed across kombucha products (Figure 4B). Sparse partial least squares discriminant (sPLS-DA)—a method for feature selection and classification—was used to identify 14 of these metabolites that formed two derived axes that could differentiate the kombucha products (Figure 4C). The first axis (X-variate 1) consisted of nine dipeptides and separated four products (E1, E2, B, D) from the others (Figure 4D). Dipeptides have been reported to rise markedly during fermentation of black tea and may represent a marker of the black tea base of kombucha products and/or their breakdown during kombucha fermentation [29]. The second axis (X-variate 2) consisted of 5 chemicals that are associated with black tea fermentation (5-O-Caffeoylshikimic acid), rooibos tea (nothofagin), and plants (7-hydroxy-8-methoxycoumarin and viburtinal) [30,31,32]. This axis, which may reflect the properties of the tea base, separated three products from the others (E1, E2, A2). 

Procrustes analysis was performed to assess for a relationship between variation in microbial gene content and metabolites across kombucha products. Differences in microbial gene content across kombucha products were significantly associated with the differences in metabolomics profiles (*p* = 3 × 10^−5^) (Figure 4E). Partial correlation analysis was performed to identify specific microbe-metabolite correlations underlying this global association after controlling for kombucha product. There were 83 microbe-metabolite correlations with false discovery rate less than 0.1, representing 32 MetaCyc pathways and 62 metabolites (Figure 4F). 

### 3.3. Ethanol, Acidity, and Caffeine Content of Kombucha

**Figure 5:**
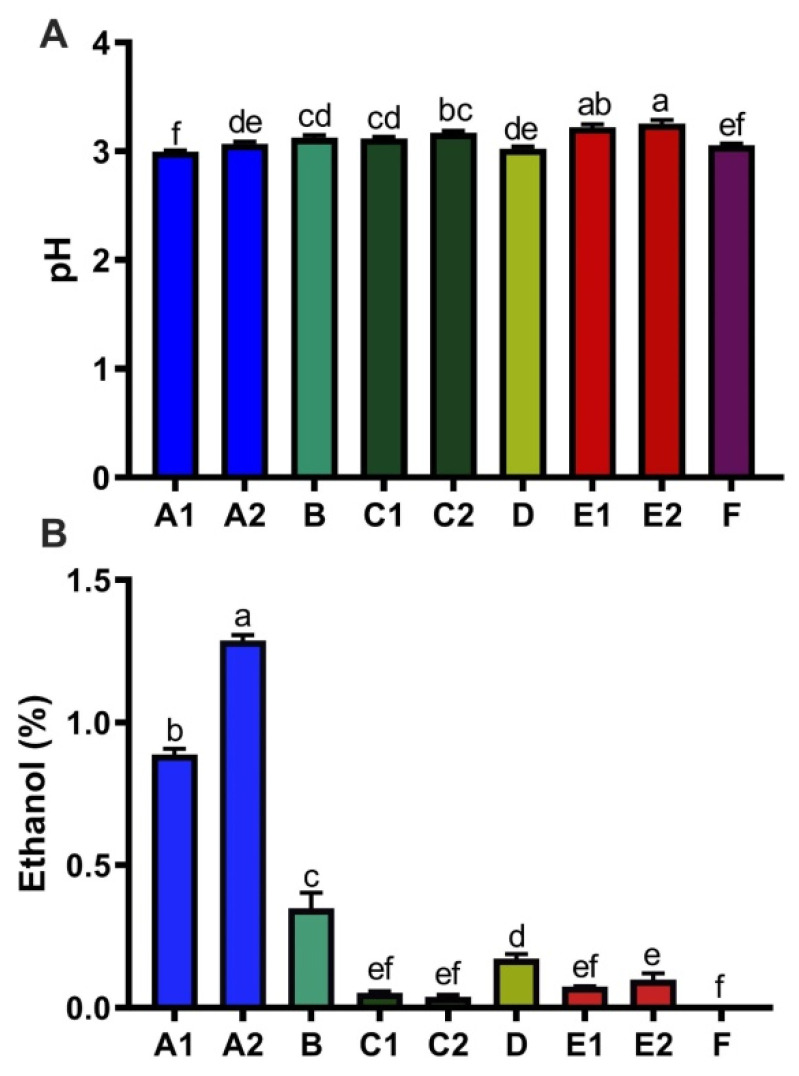
Variation in ethanol content and pH across kombucha products. pH (**A**) and ethanol (**B**) are shown for the kombucha products. Data presented as mean ± SEM (*n* = 3). Means in a column without a common letter differ; *p* < 0.05. Ethanol measurements for Product C1 had *p* < 0.05 by the Shapiro–Wilk test of normality.

**Figure 6:**
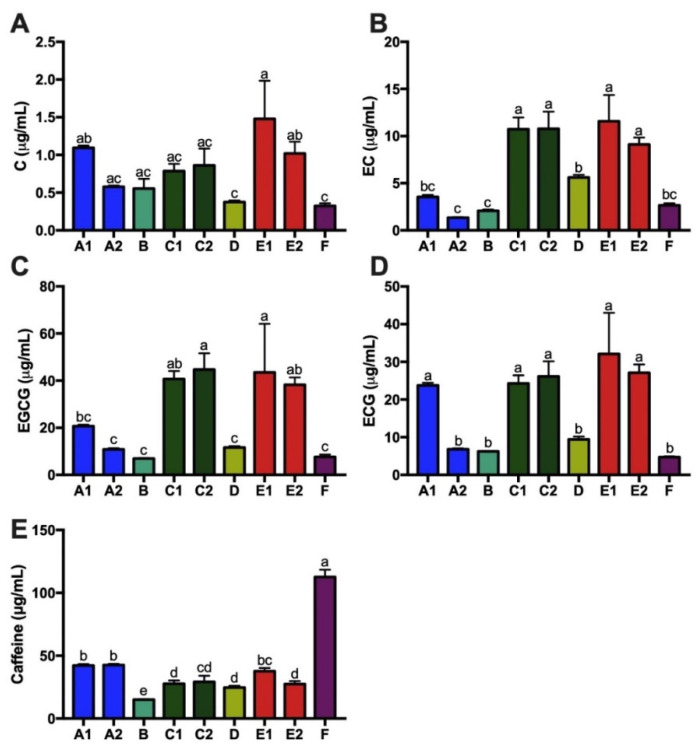
Tea catechin levels and caffeine content vary across kombucha products. Green tea catechins (**A**) C, (**B**) EC, (**C**) EGCG, (**D**) ECG, and (**E**) caffeine were measured in the nine kombucha products. Data are presented as mean ± SEM (*n* = 3). Means in a column without a common letter differ; *p* < 0.05. Products A1 (C, caffeine), A2 (EC), B (ECG), D (EC), and F (C, caffeine) had *p* < 0.05 by the Shapiro–Wilk test of normality for the indicated catechin or caffeine.

Fermentation of kombucha decreases pH due to production of organic acids [5]. The nine kombucha products had small variations in acidity with pH range from 3–3.2 (Figure 5A). One major product of fermentation is ethanol, which was detected in eight out of nine kombocha products. Ethanol content of these nine kombucha products showed large variation, ranging from 0% (F) to 1.29% (A2) (Figure 5B). The kombucha products also varied greatly in concentrations of tea catechins (C, EC, EGCG and ECG) and caffeine (Figure 6). Levels of C, EC, EGCG and ECG among these nine kombucha products were highly correlated, with correlation coefficients ranges from 0.599–0.952 (Supplementary Table S2). Ranking of total tea catechins (µg/mL) in the nine kombucha products was E1 (88.6(34.8)) > C2 (82.5(13)) > C1 (76.5(6.9)) > E2 (75.4(6.3)) > A1 (49.1(2.2)) > D (27(1.4)) > A2 (19.6(1.1)) > B (15.9(0.4)) > F (15.3(1.3)). Ranking of caffeine (µg/mL) was F (112.6(5.8)) > A2 (42.5(1.6)) > A1 (42.2(1.8)) > E1 (37.7(2.4)) > C2 (29.2(4.9)) > C1 (27.7(2.7)) > E (227.4(2.3)) > D (24.7(1.3)) > B (15.1(0.3)). 

### 3.4. Tea Catechins, Antioxidant Capacity and Total Tea Polyphenols of Kombucha Products

**Figure 7:**
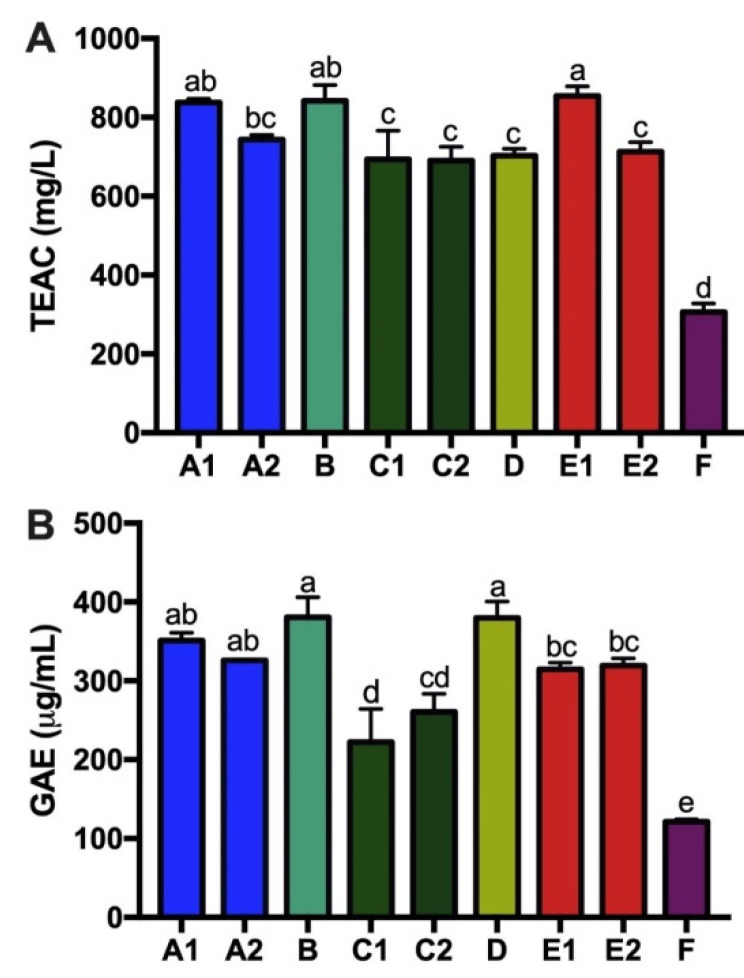
Antioxidant capacity differs across kombucha products. TEAC (**A**) and GAE (**B**) are shown as mean ± SEM (*n* = 3). Means in a column without a common letter differ; *p* < 0.05.

TEAC and GAE assays were conducted to determine the antioxidant properties and total tea polyphenols of the nine kombucha products. Results from TEAC (antioxidant capacity) and GAE (total tea polyphenols) showed a significant correlation (Pearson correlation 0.81, *p* = 0.00) between these two assays. In both assays, F had significantly lower antioxidant capacity compared to all other kombucha products with only 306.2 mg/L TEAC and 121.6 mg/L GAE. Product B was consistently highest for both assays with 842.0 mg/L TEAC and 380 mg/L GAE, showing almost a three-fold higher TEAC compared with F (Figure 7). 

## 4. Discussion

Kombucha as a functional food represents a quickly growing sector of the food and beverage industry. Many beverage companies are entering the market and generating new kombucha products with variable fermentation processes and starter ingredients, potentially impacting the chemical and microbial profiles of the final products and thereby their health effects. Here, we selected nine commercial kombucha products and analyzed their microbial and chemical profiles. These nine kombucha products were prepared from green tea (C1, C2), black tea (B) or a mixture of green/black tea (A1, A2, D, E1, E2, K) (Table 1). In addition to different tea bases, these products varied in the addition of probiotics to the kombucha. Our microbial composition analysis demonstrated that in some cases (A1 and F; E2 to a lesser extent) the final kombucha products were dominated by one of these probiotics, *Bacillus coagulans*. *Bacillus coagulans* has many known effects on health and has been commonly added to foods due to its heat resistance [18]. Many studies have reported that acetic acid bacteria were more commonly found in kombucha products than lactic bacteria, with a study of over 100 commercial kombucha starter cultures reporting that *Komagataeibacter* was most prevalent and abundant [1,33,34,35]. Lactic acid bacteria (primarily *Lactobacillus*) are less commonly used in kombucha fermentation to enhance its biological function or added as probiotics [36]. Our results showed that four out of nine commercial kombucha products (B, C1, C2 and D) were dominated by *Lactobacillus.*

The high abundance of gut-derived microbes such as *E. coli* and *B. thetaiotamicron* in some kombucha samples was surprising given the literature on antimicrobial activities of kombucha, including against *E. coli* [37,38]. However, it has been reported that *E. coli* can survive the kombucha fermentation process in some settings, supporting the possibility that gut microbes could establish a place in kombucha cultures under certain conditions [39]. Our findings that enteric microbes were present in all kombucha products is consistent with at least one previous study which found low levels of gut microbes in kombucha batches after 14 days of fermentation, including *Akkermansiaceae*, *Bifidobacteriaceae*, *Enterobacteriaceae*, and *Bacteroidaceae* [40]. The potential for these microbes to have benefit for consumers is exemplified by the high abundance of *Akkermansia muciniphila*, up to 4.3% in one sample of A2. This microbe has been reported in many human and animal model studies to be associated with improved metabolic health, including one randomized clinical trial, and to our knowledge has not been previously shown to exist in any food product [41]. However, since we do not know the absolute count of these enteric bacteria or what fraction remain viable, further investigation is required to determine whether the presence of these enteric bacteria has any health impact. Another limitation is the possibility that there was contamination during the extraction and/or sequencing steps which introduced a signal for enteric bacteria. We did not find evidence of this based upon absence of detected enteric bacteria in a blank extraction control as well as in a sample of unfermented tea base provided by a kombucha manufacturer (other than *B. thetaiotamicron*). In contrast, we observed low abundances of enteric microbes in a sample of unfermented tea base with kombucha starter culture (data not shown).

Consistent with prior studies, we found that *Brettanomyces* was the predominant yeast in five of the kombucha products [35,42]. However, the three other common yeast genera present in kombucha cultures (*Zygosaccharomyces*, *Lachancea* and *Starmerella*) were largely undetected in the nine kombucha products [35]. Instead, two products and one sample of a third product showed predominance of *Cyberlindnera jadinii*. This yeast is also known as torula and is commonly used in food products. It has been reported once in the literature to be present in kombucha [3]. The remaining two kombucha products had predominance of *Trigonopsis variabilis* or *Issatchenkia orientalis*; the latter has previously been reported in at least one kombucha sample [42].

We further identified large differences in chemical composition across kombucha products both at a global level with untargeted metabolomics and in concentrations of tea polyphenols and antioxidants. The kombucha products could be differentiated by dipeptides and tea-derived chemicals that may reflect differences in the tea base used across the products. We observed significant correlation of global metabolomics profiles with microbial gene content, suggesting that variability in the chemical composition of kombucha products may also be partially attributable to the distinct metabolic capabilities of the microbes present in various kombucha products. This was supported by the associations of many bacterial pathways with specific metabolites in kombucha. One limitation is that the kombucha products varied in their ingredients, energy content, and use of green vs. black tea base which would contribute to the observed differences. The relationships of microbes with chemical composition are likely to be greatly impacted by differences in the fermentation process and tea base across the kombucha products and would require further information on these factors to elucidate. As the samples of each kombucha product were obtained at the same time and were likely manufactured in the same batch, it is also unclear how much variability exists between batches of the same commercial kombucha product.

Antioxidant potential (TEAC) is of great interest to consumers and was found to vary across the nine kombucha products, highly correlated with variation in total tea polyphenols (GAE). However, the tea catechins, known for their antioxidant capability, did not correlate with antioxidant potential and total tea polyphenols. The four tea catechins measured in this study, C, EC, ECG and EGCG, are mainly green tea catechins, which oxidize to theaflavins during black tea production. Theaflavins and catechins provide equal antioxidant potential [43]. It is therefore possible that other tea polyphenols we did not measure, such as theoflavines, theobromines and gallates [44], are important contributors to the total antioxidant capacity. Storage conditions after initial production may have also affected the observed profiles, as it has been recently demonstrated that refrigeration of homemade kombucha for longer than 4 months results in reduced polyphenol content and antioxidant properties [45].

## 5. Conclusions

Our results demonstrate that commercial kombucha products differ from each other on multiple levels that interest the consumer and are potentially relevant for their health effects, including tea polyphenols, chemical profiles, and microbes.

## Figures and Tables

**Table 1:** List of kombucha products.

Product	Ingredients Label	Nutrition (per 100 mL)	Expiration
A1	**Probiotics**: kombucha culture, Bacillus Coagulans GBI-306086 (4 billion organisms), *S. Boulardii* (4 billion organisms), *Lactobacillus bacteria* (4 billion) **Tea**: black tea, green tea **Flavors**: kiwi juice, ginger juice	Calories: 10.6 Carbohydrate: 2.54 g, Added Sugar: 0 g Sodium: 2.11 mg Lactic acid: 21.1 mg, Acetic acid: 15.9 mg, Glucuronic acid: 296.0 mg, Gluconic acid: 137.4 mg	5/6/2021
A2	**Probiotics**: kombucha culture, *Lactobacillus bacteria* (4 billion organisms), *S. Boulardii* (4 billion organisms) **Tea**: black tea, green tea **Sugar**: cane sugar	Calories: 12.5 Carbohydrate: 3.33 g, Added Sugar: 2.08 g Sodium: 2.08 g Lactic acid: 20.83 mg, Acetic acid: 15.6 mg, Glucuronic acid: 291.7 mg, Gluconic acid: 135.4 mg Polyphenols: 2.1 mg	4/14/2021
B	**Probiotics**: kombucha culture **Tea**: black tea **Sugar**: cane sugar **Flavors**: ginger, lemongrass, orange peel, spearmint, peppermint	Calories: 12.7 Carbohydrate: 2.56 g, Added Sugar: 1.83 g Sodium: 3.67 mg	8/12/2021
C1	**Probiotics**: kombucha culture **Tea**: green tea **Sugar**: cane sugar **Flavors**: dried lemon, lemon verbena, ginger juice, ginger extract	Calories: 16.9 Carbohydrate: 4.10 g, Added Sugar: 3.14 g Sodium: 0 mg	5/22/2021
C2	**Probiotics**: kombucha culture **Tea**: green tea **Sugar**: cane sugar **Flavors**: dried lemon, lemon verbena, ginger juice, ginger extract	Calories: 16.9 Carbohydrate: 3.94 g, Added Sugar: 3.38 g Sodium: 0 mg	3/9/2021
D	**Probiotics**: kombucha culture **Sugar**: cane sugar **Flavors**: ginger juice, black tea, green tea, lemon juice	Calories: 14.8 Carbohydrate: 2.96 g, Added Sugar: 1.69 g Sodium: 0 mg	7/21/2021
E1	**Probiotics**: kombucha culture, Bacillus subtilis, vitamin B12 **Tea**: green tea, black tea **Sugar**: cane sugar **Flavors**: white grape juice, apple juice, ginger juice, lemon juice	Calories: 9.6 Carbohydrate: 2.41 g, Added Sugar: 0 g Sodium: 0 mg	8/4/2021
E2	**Probiotics**: kombucha culture, Bacillus subtilis, Vitamin B12, Bacillus coagulans **Tea**: green tea, black tea **Flavors**: white grape juice, cane sugar, allulose syrup, monk fruit, ginger juice, natural flavors	Calories: 3.03 Carbohydrate: 3.03 g Added Sugar: 0 g Sodium: 0 mg	8/24/2021
F	**Probiotics**: kombucha culture, Bacillus coagulans MTCC 5856 **Tea**: black tea, green tea, black tea extract, green tea extract, black tea essence, green coffee bean extract (caffeine) **Sugar**: cane sugar **Flavors**: ginger extract, stevia leaf extract, sparkling water, natural flavor	Calories: 13.3 Carbohydrate: 3.56 g Added Sugar: 3.33 g Sodium: 3.33 mg Gluconic and acetic acid: 112.7 mg	6/3/2021

Products were labelled by manufacturer (e.g., A, B). Numbers indicate distinct products from the same manufacturer (e.g., 1, 2).

## Data Availability

The data presented in this study are available on request from the corresponding author.

## Supplementary Materials

The following supporting information can be downloaded at: https://www.mdpi.com/article/10.3390/nu14030670/s1, Table S1: Data distributions, Table S2: Spearman correlation between tea catechins, Figure S1: Data distributions shown as boxplots.

## References

[1] Kim J., Adhikari K. (2020). Current Trends in Kombucha: Marketing Perspectives and the Need for Improved Sensory Research. Beverages.

[2] Chakravorty S., Bhattacharya S., Chatzinotas A., Chakraborty W., Bhattacharya D., Gachhui R. (2016). Kombucha tea fermentation: Microbial and biochemical dynamics. Int. J. Food Microbiol..

[3] Jayabalan R., Malbaša R.V., Lončar E.S., Vitas J.S., Sathishkumar M. (2014). A Review on Kombucha Tea—Microbiology, Composition, Fermentation, Beneficial Effects, Toxicity, and Tea Fungus. Compr. Rev. Food Sci. Food Saf..

[4] Vina I., Semjonovs P., Linde R., Denina I. (2014). Current Evidence on Physiological Activity and Expected Health Effects of Kombucha Fermented Beverage. J. Med. Food.

[5] Mousavi S.M., Hashemi S.A., Zarei M., Gholami A., Lai C.W., Chiang W.H., Omidifar N., Bahrani S., Mazraedoost S. (2020). Recent Progress in Chemical Composition, Production, and Pharmaceutical Effects of Kombucha Beverage: A Complementary and Alternative Medicine. Evid.-Based Complement. Altern. Med..

[6] Yang D.-J., Hwang L.S., Lin J.-T. (2007). Effects of different steeping methods and storage on caffeine, catechins and gallic acid in bag tea infusions. J. Chromatogr. A.

[7] Lin Y.S., Tsai Y.J., Tsay J.S., Lin J.K. (2003). Factors affecting the levels of tea polyphenols and caffeine in tea leaves. J. Agric. Food Chem..

[8] Graham H.N. (1992). Green tea composition, consumption, and polyphenol chemistry. Prev. Med..

[9] Khan N., Mukhtar H. (2007). Tea polyphenols for health promotion. Life Sci..

[10] Khan N., Mukhtar H. (2018). Tea Polyphenols in Promotion of Human Health. Nutrients.

[11] Dufresne C., Farnworth E. (2000). Tea, Kombucha, and health: A review. Food Res. Int..

[12] Lee K.W., Lee H.J., Lee C.Y. (2002). Antioxidant activity of black tea vs. green tea. J. Nutr..

[13] Gramza-Michalowska A. (2014). Caffeine in Tea Camellia Sinensis—Content, Absorption, Benefits and Risks of Consumption. J. Nutr. Health Aging.

[14] Heber D., Zhang Y.J., Yang J.P., Ma J.E., Henning S.M., Li Z.P. (2014). Green Tea, Black Tea, and Oolong Tea Polyphenols Reduce Visceral Fat and Inflammation in Mice Fed High-Fat, High-Sucrose Obesogenic Diets. J. Nutr..

[15] Chan E.W.C., Soh E.Y., Tie P.P., Law Y.P. (2011). Antioxidant and antibacterial properties of green, black, and herbal teas of Camellia sinensis. Pharm. Res..

[16] Sengun I., Kirmizigul A. (2020). Probiotic Potential of Kombucha. J. Clin. Gastroenterol..

[17] Vargas B.K., Fabricio M.F., Ayub M.A.Z. (2021). Health effects and probiotic and prebiotic potential of Kombucha: A bibliometric and systematic review. Food Biosci..

[18] Konuray G., Erginkaya Z. (2018). Potential Use of Bacillus coagulans in the Food Industry. Foods.

[19] Blanc P.J. (1996). Characterization of the tea fungus metabolites. Biotechnol. Lett..

[20] Villarreal-Soto S.A., Beaufort S., Bouajila J., Souchard J.-P., Taillandier P. (2018). Understanding Kombucha Tea Fermentation: A Review. J. Food Sci..

[21] SPINS TOTAL US $ SALES (MULO, CONVENIENCE, NATURAL) L52W ENDING 12.27.20. https://www.spins.com.

[22] Singleton V.L., Esau P. (1969). Phenolic substances in grapes and wine, and their significance. Adv. Food Res. Suppl..

[23] Henning S.M., Zhang Y., Seeram N.P., Lee R.P., Wang P., Bowerman S., Heber D. (2011). Antioxidant capacity and phytochemical content of herbs and spices in dry, fresh and blended herb paste form. Int. J. Food Sci. Nutr..

[24] Beghini F., McIver L.J., Blanco-Miguez A., Dubois L., Asnicar F., Maharjan S., Mailyan A., Manghi P., Scholz M., Thomas A.M. (2021). Integrating taxonomic, functional, and strain-level profiling of diverse microbial communities with bioBakery 3. Elife.

[25] McMurdie P.J., Holmes S. (2013). phyloseq: An R package for reproducible interactive analysis and graphics of microbiome census data. PLoS ONE.

[26] Walters W., Hyde E.R., Berg-Lyons D., Ackermann G., Humphrey G., Parada A., Gilbert J.A., Jansson J.K., Caporaso J.G., Fuhrman J.A. (2016). Improved Bacterial 16S rRNA Gene (V4 and V4-5) and Fungal Internal Transcribed Spacer Marker Gene Primers for Microbial Community Surveys. mSystems.

[27] Nilsson R.H., Larsson K.H., Taylor A.F.S., Bengtsson-Palme J., Jeppesen T.S., Schigel D., Kennedy P., Picard K., Glockner F.O., Tedersoo L. (2019). The UNITE database for molecular identification of fungi: Handling dark taxa and parallel taxonomic classifications. Nucleic Acids Res..

[28] Le Cao K.A., Boitard S., Besse P. (2011). Sparse PLS discriminant analysis: Biologically relevant feature selection and graphical displays for multiclass problems. BMC Bioinform..

[29] Xue J., Liu P., Guo G., Wang W., Zhang J., Wang W., Le T., Yin J., Ni D., Jiang H. (2022). Profiling of dynamic changes in non-volatile metabolites of shaken black tea during the manufacturing process using targeted and non-targeted metabolomics analysis. LWT.

[30] Joubert E. (1996). HPLC quantification of the dihydrochalcones, aspalathin and nothofagin in rooibos tea (Aspalathus linearis) as affected by processing. Food Chem..

[31] Godeau R. P., Rossi J. C., Fouraste I. (1977). Methyl-4-formyl-7 cyclopenta(c)pyrane isolated by acid hydrolysis from Viburnum tinus. Phytochemistry.

[32] Barua N., Sharma R.P., Madhusudanan K.P., Thyagarajan G., Herz W. (1980). Coumarins in Artemisia Caruifolia. Phytochemistry.

[33] De Roos J., De Vuyst L. (2018). Acetic acid bacteria in fermented foods and beverages. Curr. Opin. Biotechnol..

[34] May A., Narayanan S., Alcock J., Varsani A., Maley C., Aktipis A. (2019). Kombucha: A novel model system for cooperation and conflict in a complex multi-species microbial ecosystem. PeerJ.

[35] Harrison K., Curtin C. (2021). Microbial Composition of SCOBY Starter Cultures Used by Commercial Kombucha Brewers in North America. Microorganisms.

[36] Nguyen N.K., Dong N.T., Nguyen H.T., Le P.H. (2015). Lactic acid bacteria: Promising supplements for enhancing the biological activities of kombucha. Springerplus.

[37] Sreeramulu G., Zhu Y., Knol W. (2000). Kombucha fermentation and its antimicrobial activity. J. Agric. Food Chem..

[38] Bhattacharya D., Bhattacharya S., Patra M.M., Chakravorty S., Sarkar S., Chakraborty W., Koley H., Gachhui R. (2016). Antibacterial Activity of Polyphenolic Fraction of Kombucha against Enteric Bacterial Pathogens. Curr. Microbiol..

[39] Brewer S.S., Lowe C.A., Beuchat L.R., Ortega Y.R. (2021). Survival of Salmonella and Shiga Toxin-Producing Escherichia coli and Changes in Indigenous Microbiota during Fermentation of Home-Brewed Kombucha. J. Food Prot..

[40] Gaggìa F., Baffoni L., Galiano M., Nielsen D., Jakobsen R., Castro-Mejía J., Bosi S., Truzzi F., Musumeci F., Dinelli G. (2018). Kombucha Beverage from Green, Black and Rooibos Teas: A Comparative Study Looking at Microbiology, Chemistry and Antioxidant Activity. Nutrients.

[41] Depommier C., Everard A., Druart C., Plovier H., Van Hul M., Vieira-Silva S., Falony G., Raes J., Maiter D., Delzenne N.M. (2019). Supplementation with Akkermansia muciniphila in overweight and obese human volunteers: A proof-of-concept exploratory study. Nat. Med..

[42] Mayser P., Fromme S., Leitzmann C., Grunder K. (1995). The yeast spectrum of the ‘tea fungus Kombucha’. Mycoses.

[43] Leung L.K., Su Y., Chen R., Zhang Z., Huang Y., Chen Z.Y. (2001). Theaflavins in black tea and catechins in green tea are equally effective antioxidants. J. Nutr..

[44] Dutta H., Paul S.K. (2019). Kombucha Drink: Production, Quality, and Safety Aspects.

[45] La Torre C., Fazio A., Caputo P., Plastina P., Caroleo M.C., Cannataro R., Cione E. (2021). Effects of Long-Term Storage on Radical Scavenging Properties and Phenolic Content of Kombucha from Black Tea. Molecules.

