# Supplementary material for: Microbial and Chemical Profiles of Commercial Kombucha Products
Source: Nutrients. 2022 Feb 5;14(3):670. doi: 10.3390/nu14030670 (PMC8838605; doi:10.3390/nu14030670)
Supplement: Supplementary file 1 [file nutrients-14-00670-s001.zip › nutrients-1572293-supplementary-done.pdf]

nutrients

MDPI

Table S1: Data distributions

	Mean ± SD	Median	Minimum	Maximum	Shapiro-Wilk (P value)
TEAC (mg/dL)					
**A1**	837.8±16.7	841.9	819.5	852.1	0.59
**A2**	744.1±19.4	734.4	731.5	766.5	0.14
**B**	842±39.9	827.3	811.5	887.1	0.38
**C1**	693.9±72.3	694.5	621.3	765.9	0.99
**C2**	690.3±34.8	687.3	657.1	726.4	0.86
**D**	703.3±17.3	703.2	686	720.6	0.99
**E1**	854.6±24.4	856.5	829.3	877.9	0.87
**E2**	712.7±24.6	712.7	688.2	737.3	1.00
**F**	306.2±21.8	314.8	281.4	322.4	0.33
GAE (µg/mL)					
**A1**	351.1±17.2	346.1	337	370.2	0.51
**A2**	325.9±2.6	326.7	323	327.9	0.45
**B**	380.6±25.3	368.9	363.3	409.7	0.21
**C1**	222.3±42	207.2	190	269.8	0.39
**C2**	260.6±23.1	270.8	234.2	276.9	0.25
**D**	379.3±20.7	377.3	359.7	401	0.84
**E1**	314.8±8.4	318.1	305.2	321	0.33
**E2**	319.4±9.2	320.2	309.9	328.2	0.86
**F**	121.6±2.7	121.3	119.1	124.4	0.81
C (µg/mL)					
**A1**	1.1±0.1	1.1	1.1	1.2	0.00
**A2**	0.6±0	0.6	0.6	0.6	
**B**	0.5±0.2	0.5	0.4	0.7	0.64
**C1**	0.8±0.1	0.8	0.7	0.9	1.00
**C2**	0.9±0.3	0.9	0.6	1.1	0.78
**D**	0.4±0	0.4	0.4	0.4	
**E1**	1.5±0.5	1.6	0.9	1.9	0.57
**E2**	1±0.2	1.0	0.9	1.2	0.64
**F**	0.3±0.1	0.3	0.3	0.4	0.00
EC (µg/mL)					
**A1**	3.5±0.4	3.7	3.1	3.8	0.25
**A2**	1.3±0.1	1.4	1.2	1.4	0.00
**B**	2.1±0.2	2.1	1.9	2.2	0.64
**C1**	10.7±1.2	10.6	9.6	12	0.82
**C2**	10.8±1.8	8.8	8.8	12.3	0.54
**D**	5.6±0.3	5.4	5.4	5.9	0.00

**E1**	11.6±2.8	12.2	8.5	14	0.62
**E2**	9.1±0.8	9.1	8.4	9.9	0.93
**F**	2.6±0.2	2.7	2.4	2.8	0.46
EGCG ((µg/mL)					
**A1**	20.7±1	20.5	19.9	21.8	0.60
**A2**	10.9±0.9	10.9	10	11.7	0.94
**B**	7±0.2	7.0	6.8	7.1	0.64
**C1**	40.7±3.4	40.9	37.2	43.9	0.88
**C2**	44.7±6.9	47.1	36.9	50.1	0.42
**D**	11.6±0.6	11.9	10.9	12	0.16
**E1**	43.4±20.6	51.1	20.1	59.1	0.37
**E2**	38.2±3.2	37.9	35.2	41.6	0.83
**F**	7.7±1	7.2	7	8.8	0.19
ECG (µg/mL)					
**A1**	23.8±1.1	23.8	22.7	24.9	1.00
**A2**	6.8±0.4	6.9	6.4	7.2	0.73
**B**	6.3±0.1	6.3	6.2	6.3	0.00
**C1**	24.3±2.2	24.2	22.1	26.5	0.95
**C2**	26.2±4	27.3	21.7	29.5	0.53
**D**	9.4±0.8	9.1	8.8	10.3	0.36
**E1**	32.1±10.9	36.1	19.8	40.4	0.38
**E2**	27.1±2.3	27.0	24.9	29.4	0.93
**F**	4.7±0.2	4.7	4.5	4.9	1.00
Caffeine (µg/mL)					
**A1**	42.2±1.9	43.3	40.1	43.3	0.00
**A2**	42.6±1.6	42.5	41	44.2	0.93
**B**	15.1±0.3	15.1	14.8	15.4	1.00
**C1**	27.6±2.7	28.0	24.8	30.1	0.77
**C2**	29.2±4.9	30.4	23.8	33.4	0.59
**D**	24.7±1.3	24.7	23.4	26	1.00
**E1**	37.7±2.5	39.0	34.9	39.3	0.12
**E2**	27.4±2.4	27.3	25.1	29.8	0.93
**F**	112.6±5.8	109.3	109.2	119.3	0.02
pH					
**A1**	3±0	3.0	3	3	1.00
**A2**	3.1±0	3.1	3.1	3.1	1.00
**B**	3.1±0	3.1	3.1	3.2	0.46
**C1**	3.1±0	3.1	3.1	3.1	0.64
**C2**	3.2±0	3.2	3.2	3.2	0.64
**D**	3±0	3.0	3	3	0.46
**E1**	3.2±0	3.2	3.2	3.3	0.78

**E2**	3.3±0	3.3	3.2	3.3	0.64
**F**	3.1±0	3.1	3	3.1	0.64
ETOH (%)					
**A1**	0.9±0	0.9	0.9	0.9	0.54
**A2**	1.3±0	1.3	1.3	1.3	0.84
**B**	0.4±0.1	0.4	0.3	0.4	0.36
**C1**	0.1±0	0.0	0.1	0.1	0.00
**C2**	0±0	0.0	0	0.1	1.00
**D**	0.2±0	0.2	0.2	0.2	0.64
**E1**	0.1±0	0.1	0.1	0.1	
**E2**	0.1±0	0.1	0.1	0.1	0.46
**F**	0±0	0.0	0	0	
Fungal Shannon index					
**A1**	0.7±0	0.7	0.7	0.7	0.19
**A2**	0.6±0	0.6	0.6	0.6	0.05
**B**	0±0	0.0	0	0	0.96
**C1**	1.7±0.1	1.7	1.7	1.8	0.65
**C2**	1.5±0	1.5	1.5	1.6	0.77
**D**	0.7±0.3	0.9	0.3	0.9	0.00
**E1**	0.7±0	0.7	0.7	0.7	0.27
**E2**	0.8±0	0.8	0.8	0.8	0.94
**F**	1.8±0.5	1.8	1.3	2.2	0.89
Microbial Shannon index					
**A1**	0.1±0.1	0.1	0.1	0.2	0.11
**A2**	1.6±0.5	1.7	1	2	0.69
**B**	0.7±0.1	0.6	0.6	0.8	0.38
**C1**	0.1±0	0.1	0.1	0.1	0.79
**C2**	0.3±0.4	0.0	0	0.7	0.00
**D**	0.2±0.1	0.2	0.1	0.3	0.99
**E1**	0.2±0.1	0.2	0.2	0.3	0.62
**E2**	0.7±0.1	0.7	0.6	0.7	0.21
**F**	0±0	0.0	0	0	0.51

Table S2: Spearman correlation between tea catechins

		C	EC	EGCG	ECG
Correlation Coefficient	C	1	0.599	0.783	0.875
P value		.	0.001	0	0
Correlation Coefficient	EC	0.599	1	0.91	0.849

P value		0.001	.	0	0
Correlation Coefficient	EGCG	0.783	0.91	1	0.952
P value		0	0	.	0
Correlation Coefficient	ECG	0.875	0.849	0.952	1
P value		0	0	0	.

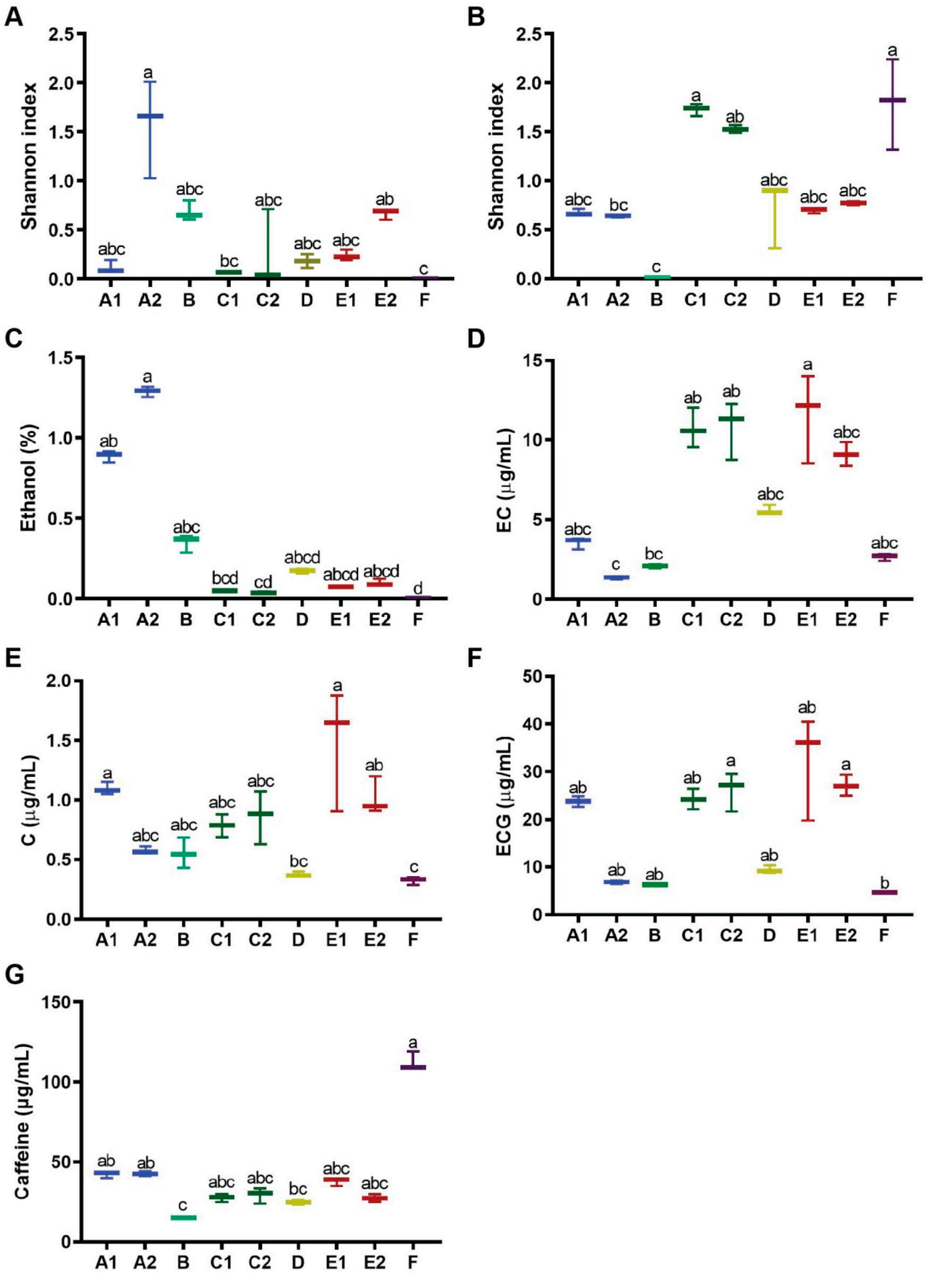

Figure S1. Data distributions are shown as boxplots (lines represent median, upper and lower bars represent maximum and minimum) for measures which had at least one out of nine kombucha products with $p<0.05$ by the Shapiro-Wilk test (a test of normality of the distribution): bacterial Shannon index (**A**), fungal Shannon index (**B**), ethanol (**C**), EC (**D**), C (**E**), ECG (**F**), and caffeine (**G**). Means in a column without a common letter differ by the non-parametric Kruskal-Wallis test with post-hoc Dunn test; $p < 0.05$.
